# Supplementary material for: Splice-Junction-Based Mapping of Alternative Isoforms in the Human Proteome
Source: Cell Rep. Author manuscript; Available in PMC 2020 Jan 15. (PMC6961840; doi:10.1016/j.celrep.2019.11.026)
Supplement: 2 [file NIHMS1546469-supplement-2.zip › DF1/PXD009737/TestisGluC/TestisGluC_29_CCNDBP1_SFRAPLASVPPASRPLFLPLSPTE.pdf]

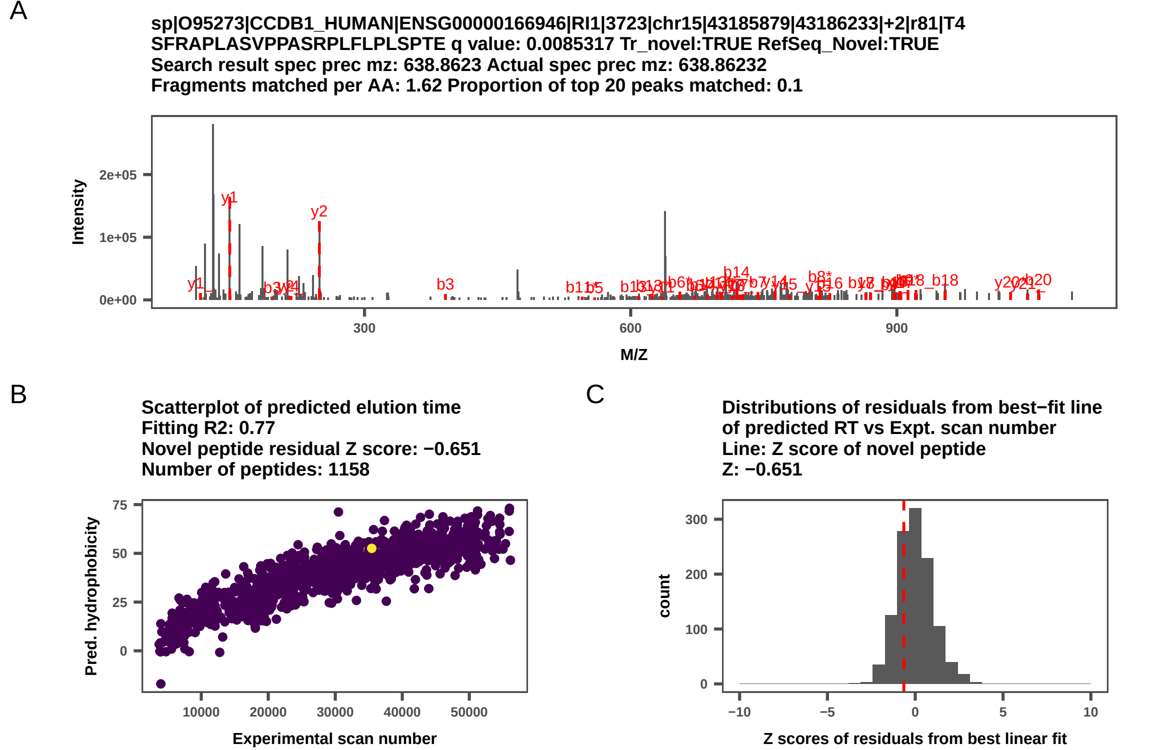
A
sp|O95273|CCDB1_HUMAN|ENSG00000166946|RI1|3723|chr15|43185879|43186233|+2|r81|T4
SFRAPLASVPPASRPLFLPLSPTE q value: 0.0085317 Tr_novel:TRUE RefSeq_Novel:TRUE
Search result spec prec mz: 638.8623 Actual spec prec mz: 638.86232
Fragments matched per AA: 1.62 Proportion of top 20 peaks matched: 0.1
Intensity
2e+05
1e+05
0e+00
y1
y2
b3
b14
b8*
b18
300
600
900
M/Z
B
Scatterplot of predicted elution time
Fitting R2: 0.77
Novel peptide residual Z score: −0.651
Number of peptides: 1158
Pred. hydrophobicity
75
50
25
0
10000
20000
30000
40000
50000
Experimental scan number
C
Distributions of residuals from best−fit line
of predicted RT vs Expt. scan number
Line: Z score of novel peptide
Z: −0.651
count
300
200
100
0
−10
−5
0
5
10
Z scores of residuals from best linear fit
